# Microcirculation and Cardiovascular Diseases

**DOI:** 10.5935/abc.20180149

**Published:** 2018-08

**Authors:** Eduardo Tibiriçá, Andrea De Lorenzo, Gláucia Maria Moraes de Oliveira

**Affiliations:** 1 Programa de Pós-Graduação em Cardiologia da Universidade Federal do Rio de Janeiro, Rio de Janeiro, RJ – Brazil; 2 Mestrado profissional em Ciências Cardiovasculares do Instituto Nacional de Cardiologia, Rio de Janeiro, RJ – Brazil

**Keywords:** Microcirculation/physiology, Humans, Cardiovascular Diseases/physiopathology, Endothelium, Vascular/pysiology, Vasodilatation/physiology, Risk Factors, Diagnostic Imaging

Human microcirculation has some aspects that make it unique in its capacity to adjust the supply of oxygen and nutrients to the metabolic demands of all cells throughout the body by adjusting vascular tone and releasing different vasoactive substances.^1^ Endothelium-dependent vasodilation response in humans may vary according to age, gender, the vascular bed involved and the presence of atherosclerotic disease. Vascular smooth muscle cells undergo different hyperpolarization and relaxation when exposed to nitric oxide (NO), prostacyclin and endothelium-derived hyperpolarizing factors (EDHF), among others, as a result of the factors described above.^2^

In the last decade, the importance of assessing microvascular function has become evident in research on the pathophysiology of cardiovascular disease and cardiovascular risk stratification.^3^ In this context, cutaneous microcirculation has been considered an accessible and representative vascular bed for assessing microvascular reactivity.^4^ Indeed, there is evidence of an association between cutaneous microvascular reactivity and the microcirculatory function in different vascular beds, concerning both the underlying mechanisms and the intensity of the endothelium-dependent vasodilation response.^4^ Therefore, assessing cutaneous microvascular reactivity has been proposed as a prognosis marker both for chronic disease and for the action of drugs related to the microvascular endothelial function.^5^

Microcirculation assessment in humans was initially performed using invasive techniques such as coronary angiography; however, the evolution of imaging techniques has made it possible to diagnose microcirculatory abnormalities in cardiovascular disease using non-invasive methods that range from ultrasound techniques such as stress echocardiogram and myocardial perfusion scintigraphy (neither directly assesing myocardial blood flow, as both are used to detect ischemia – which, in the absence of obstructive epicardial coronary disease, is considered evidence of microvascular disease) to more expensive techniques such as positron emission tomography (PET). The prognostic importance of myocardial microvascular dysfunction has been acknowledged, which has boosted studies on the subject, though it is not possible so far to directly visualize its structural abnormalities, which can only be assessed through myocardial flow or coronary flow reserve (CFR).^6^

CFR, or the ratio of hyperemic myocardial blood flow – i.e., at peak stress – to myocardial blood flow at rest,^7^ evaluates the whole hemodynamics of coronary circulation, from epicardial arteries to microcirculation, including endothelial and vascular smooth muscle function.^7^ Reduced CFR has been shown to be an independent predictor of mortality, also in patients with normal epicardial coronary arteries.^8^ CFR can be examined by PET,^9^ but because that method is not largely available, CFR exams have recently become possible by means of myocardial scintigraphy (SPECT) using solid-state gamma cameras such as the telluride-cadmium-zinc (CZT) type, which provide higher sensitivity and better temporal and spatial resolutions.^10^

As mentioned earlier, the systemic microcirculatory function can be examined using techniques for measuring the microvascular flow in the skin in a non-invasive way. In the clinical context, the methods most commonly used are based on laser light, including laser Doppler imaging and laser speckle contrast imaging (LSCI).^11^ These techniques are usually associated with physiological or pharmacological stimuli that allow assessing endothelium-dependent (or independent) microvascular reactivity.^12^ The physiological stimulus is usually forearm post-occlusive reactive hyperemia, which induces an endothelium-dependent vasodilation response. The most commonly used pharmacological stimulus is the cutaneous infusion of acetylcholine (endothelium-dependent vasodilation) or sodium nitroprusside (endothelium-independent vasodilatation), both through micro-iontophoresis.^12^

In this context, we have recently conducted a study using the LSCI technique where we demonstrated that the endothelium-dependent vasodilation response of cutaneous microcirculation is reduced in patients with early-onset coronary artery disease compared to healthy individuals^12^. In addition, the microvascular response related to vascular smooth muscle dilatation was also reduced in those patients, in parallel with significant increases of carotid intima-media thickness.^12^ In another study, we demonstrated that the systemic microvascular endothelial function is similarly compromised in patients with ischemic heart disease or chronic Chagas heart disease.^13^

Recently, we adapted the LSCI technique for noninvasive assessment of penile microvascular reactivity.^14^ In that study, we demonstrated that LSCI can be used to assess the effects of type 5 phosphodiesterase inhibitors on penile microcirculation in patients with hypertension and erectile dysfunction.^14^

Microcirculation rarefaction has been associated with cardiovascular and metabolic diseases, including hypertension, diabetes, obesity and metabolic syndrome.^15^

Change in the microvascular function in the skin has also been shown to correlate with an increased risk of coronary artery disease.^16^ In addition, the rarefaction of microcirculation in capillary beds is related to target organ damage, which was suggested by the existence of an association between myocardial disease and the reduction of capillary density, as well as another association between left ventricular hypertrophy and cutaneous microvascular dysfunction, regardless of the level of systemic arterial pressure.^17^^,^^18^ In a recent study, we demonstrated that cutaneous capillary density, as well as endothelium-dependent capillary recruitment, are reduced in patients with early-onset coronary artery disease.^19^

Therefore, the early detection of subclinical cardiovascular disease through the assessment of microcirculatory density and reactivity non-invasive techniques could represent an opportunity for early intervention, and consequently, prevention of cardiovascular events.^20^ Moreover, microcirculation assessment could be useful to evaluate the chronic effects of cardiovascular drugs, making it attractive not only in research contexts but also in clinical practice.

Thus, we believe that microcirculation assessment will be increasingly employed in cardiovascular practice, both for diagnostic and prognostic purposes and for testing novel therapeutic interventions.
